# Implementation of Ultrasound in Preclinical Education at Osteopathic Medical Schools: A Scoping Review

**DOI:** 10.7759/cureus.39329

**Published:** 2023-05-22

**Authors:** Marianne R Scotti, Dakota C Davis, Riya Patel, Seddrick Weekes, Thomas McNary, Julia Alexander

**Affiliations:** 1 Medical School, Alabama College of Osteopathic Medicine, Dothan, USA; 2 Anatomy, Alabama College of Osteopathic Medicine, Dothan, USA; 3 Radiology, Alabama College of Osteopathic Medicine, Dothan, USA

**Keywords:** ultrasound, anatomy, osteopathic medical school, medical school education, ultrasound education, scoping study

## Abstract

Ultrasound (US) is recognized as a practical and safe form of medical imaging that utilizes ultrasound waves to develop images for diagnostic and procedural purposes. The clinical use of US has dramatically increased over recent years, secondary to the ease of use, portability, and functionality of US. The success of point-of-care ultrasound implementation into residency curricula has further underscored the importance of US education and its potential for use earlier in medical instruction. Osteopathic medical education places a significant emphasis on anatomy, thus a scoping review of the literature regarding the use of US in osteopathic preclinical years is warranted.

The goal of this scoping study is to assess the current literature regarding the implementation and benefit of US instruction in preclinical osteopathic medical curricula. Four resources were utilized for the review, including PubMed, Google Scholar, JOM (formerly JAOA), and AMED, each with contiguous criteria for applicable literature. The searches were performed before the end of January 2023. Inclusion criteria for researched literature focused on osteopathic preclinical utilization of US technologies. Articles were subsequently evaluated using thematic and contextual analysis.

Of the 2,968 articles evaluated, 22 articles met the inclusion criteria. There were several themes associated with the implementation of US within osteopathic curricula, including positive student perceptions of the modality, improved learning outcomes, and adaptations of US instruction into anatomical sciences courses.

There is a need for continued research regarding US implementation in preclinical osteopathic medical school education, including within anatomical sciences. A minority of osteopathic schools have published details regarding how US has been applied in their curriculum.

## Introduction and background

Ultrasound (US) is considered a practical and safe form of medical tomography that utilizes ultrasound sound waves to generate anatomic images for diagnostic and procedural purposes. US has been utilized by physicians for the last five decades and has rapidly improved in affordability, quality, and functionality [1]. The utility of US is amplified by its ease of use and lack of health risks related to radiation [2]. Medical curricula from non-radiologic specialties expose students to ultrasonography later in their graduate and residency educations, rather than during the preclinical medical school years. The American Academy of Emergency Medicine has made a statement suggesting US be integrated into core undergraduate medical curricula [3]. Other publications also suggest that the successful implementation of ultrasonography into curricula improved physical examination skills and added a more holistic understanding of anatomical and physiological processes, all the while engaging students in an enjoyable and interactive process [2-5]. A potential reason for student engagement could be connected to technological advancements that allow US probes to connect directly to a phone or tablet. Presets in some of these new probes allow a novice to acquire functional images with less training in US physics, permitting medical students with varying amounts of experience and training to “successfully [incorporate] the use of POCUS in their clinical placements with an identification success rate of 93%” [4].

In tandem with enhanced student learning, introducing ultrasonography has proven beneficial for many medical specialties, including anesthesia, cardiology, emergency medicine, surgery and associated subspecialties, and internal medicine [5]. As an osteopathic medical education institution, we recognize US for its practicality and applicability for future use. Additionally, osteopathic medical education places a heavy focus on anatomical education, including osteopathic manipulative treatment, which makes US training a feasible and logical complement to preclinical instruction [6]. Anatomy is the cornerstone of the medical sciences, and its education is essential as an inlet to the teaching and clinical practice of medicine and surgery. It is important to add US images in teaching anatomy in parallel with other modalities to approximate anatomical facts differently. This is because US has become an almost routine investigation method and is available, cost-effective, and safe. This review will assess the available literature regarding the implementation of US into osteopathic curricula to showcase the precedence of additional research.

## Review

Search protocol

**Figure 1:**
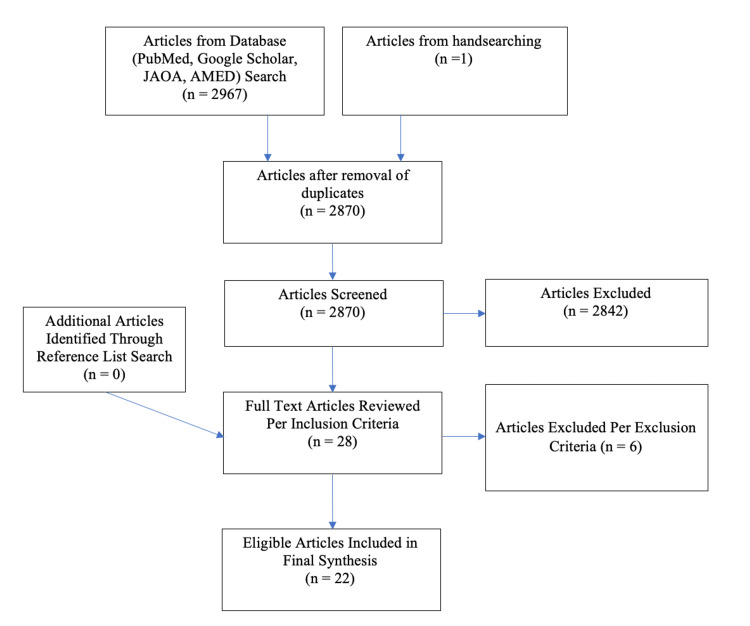
Flow diagram of literature search documenting processes utilized for research acquisition.

The comprehensive assessment of available literature for this project was initiated in May 2021 and concluded in January 2023. MS, RP, DD, SW, and TM participated in the search. All authors assisted with reviewing the articles to ensure the inclusion criteria were met. Four resources were utilized for the scoping review, namely, PubMed, Google Scholar, The Journal of Osteopathic Medicine (JOM) formerly the Journal of the American Osteopathic Association (JAOA), and the Allied and Complementary Medicine Database (AMED). The following keywords were searched in each: “Osteopathic undergraduate ultrasound,” “Osteopathic ‘medical education’ ultrasound,” “Ultrasound undergraduate osteopathic education,” “Ultrasound osteopathic education,” “Osteopathic medical school ultrasound education,” and “ultrasound preclinical osteopathic.” With each keyword, the number of articles that populated was recorded, along with the number of articles that fit the inclusion criteria and any duplicates. There was no limit to the publication date of the articles as the prevalence of US technology in medical education was considered negligible prior to 20 years ago. A master list using an Excel Spreadsheet (Microsoft) was created and used to track articles that fit into the inclusion criteria to ensure there were no duplicates in the data, as seen in Figure 1.

Inclusion and exclusion criteria

The inclusion criteria consisted of the following: educational research involving preclinical curricula, practices influencing the execution of osteopathic curricula, original articles with no duplicates, an article that was not a literature review, and research completed at an osteopathic institution. Exclusion criteria consisted of articles that were not in English/nor translated, incomplete articles, or articles not pertaining to US training of preclinical medical students. To avoid narratives, case studies, anecdotes, and small sample sizes, we also excluded publications with either a cohort size smaller than five people or an unpublished cohort size.

Google Scholar searches amassed more results than we could reliably assess. For example, one search contained more than 16,000 results. After concluding three searches of several hundred results, we determined the following procedure was acceptable. We arranged the search list according to relevance and considered results until at least 40 consecutive results were irrelevant to our search.

The aforementioned master list was used to verify at least two members of the research team reviewed each article and ensured all inclusion/exclusion criteria were properly applied. Each author added their initials to the article they reviewed. When two reviewers completed their assessment and agreed, the article was either included or excluded from the study. When there was a disagreement, an additional reviewer finalized the decision. Articles that met all the inclusion criteria were compiled into a table to summarize the results.

Unpublished articles and solitary abstracts

For the project, an unpublished solitary abstract and several poster presentations were identified. These entities were labeled as “gray literature” in the article master list. The New York Institute of Technology College of Osteopathic Medicine (NYIT-COM) literature titled “The effect of integrating ultrasound imaging into osteopathic medical student palpation training” was an example of “gray literature” utilized in this review. This was included because it focused on implementing US into a specific curriculum as an introduction to US. Upon final review in preparation for publication, this “gray literature” poster had been converted to a publication supporting similar conclusions regarding the incorporation of ultrasonography into undergraduate preclinical osteopathic curriculums [7]. We determined the published article [7] met the inclusion criteria in place of the poster.

Literature search

An electronic search of the previously cited resources (PubMed, Google Scholar, AMED, and JOM) and hand-searching resulted in 2,967 citations. After the manual removal of 97 duplicates, 2,870 citations remained to be screened (Figure 1). The 2,870 citations were assessed based on their titles and abstracts per the inclusion criteria. In the initial screening, 28 citations were retained for full-text articles and the rest were excluded per the inclusion criteria assessment. A backward search of the references from full-text articles resulted in no additional citations. Of the full-text records reviewed based upon the inclusion criteria, as stated in the methods section, six documents were excluded from the final synthesis because they met the exclusion criteria. The remaining 22 articles were included in this scoping review for final synthesis.

Ultrasound in osteopathic education among eligible studies

Of the accredited osteopathic medical schools with US in their curricula, the following nine educational institutions were responsible for the articles meeting the eligibility criteria: Kirksville College of Osteopathic Medicine (ATSU-KCOM), Arizona College of Osteopathic Medicine (AZCOM), Lake Erie College of Osteopathic Medicine Bradenton campus (LECOM-Bradenton), New York Institute of Technology College of Osteopathic Medicine (NYIT-COM), Rocky Vista University College of Osteopathic Medicine (RVU-COM), Texas College of Osteopathic Medicine (TCOM), Touro University College of Osteopathic Medicine (TUCOM), Edward Via College of Osteopathic Medicine (VCOM), and West Virginia School of Osteopathic Medicine (WVSOM).

The osteopathic medical institutions meeting the inclusion criteria of this review delivered their US curriculum in a variety of ways to their students. The integration of US into curricula occurred in many forms such as assignments, clinical courses, laboratories, examinations, application-based clinics, video modules, workshops, expert training, and peer-assisted learning. Each medical school covered a wide variety of topics and structures, as shown in Table 1. Some taught US alongside system-based curricula [8]. Two colleges used US to enhance student learning of anatomic landmarks and diagnostic skills in osteopathic manipulative medicine (OMM) [7,9,10]. AZCOM, TCOM, and TUCOM used US in their curriculum to help students learn anatomy [11-13]. US was also implemented through extracurricular activities such as student-driven free clinics and peer-assisted learning sessions for the integration of clinical knowledge at ATSU-KCOM, TCOM, and WVSOM [12,14,15]. LECOM-Bradenton and AZCOM utilized radiologists and technologists to enhance US learning and training [11,14]. Some institutions had learning opportunities through assigned clinical courses and skills labs to better expose the students to the workings of US in a clinical setting [13,14,16-18]. Embalmed cadavers were also utilized to teach needle-guided procedures such as knee arthrocentesis, subclavian vein access, PICC line insertions, central line insertions, and identification of fractures using US [14,16,19-22]. To verify the technical skills of students in US, examinations and assessments were also given [11,13,20-23].

**Table 1:** Integration of ultrasound into undergraduate medical education and objectives of ultrasound teaching. ATSU-KCOM: Kirksville College of Osteopathic Medicine; AZCOM: Arizona College of Osteopathic Medicine; LECOM-Bradenton: Lake Erie College of Osteopathic Medicine Bradenton campus; NYIT-COM: New York Institute of Technology College of Osteopathic Medicine; RVU-COM: Rocky Vista University College of Osteopathic Medicine; TCOM: Texas College of Osteopathic Medicine; TUCOM: Touro University College of Osteopathic Medicine; VCOM: Edward Via College of Osteopathic Medicine; WVSOM: West Virginia School of Osteopathic Medicine; OMM: osteopathic manipulative medicine; RUQ: right upper quadrant

Osteopathic medical school	References	Integration of ultrasound education in undergraduate years	Ultrasound topics covered
ATSU-KCOM	Kondrashova & Lockwood, 2015 [9]	OMM assignments	Musculoskeletal structures, thoracic, lumbar, sacral, craniocervical landmarks, glenohumeral joint, suprapatellar recess
	Petty et al., 2016 [23] Zoll et al., 2017 [16]	Ultrasound practical examinations	Scanning techniques, physics of ultrasound, use of ultrasound machine
	Wlodarkiewicz et al., 2020 [24]	Student-run free clinic	Application of ultrasound knowledge from clinical ultrasound courses
	Houser & Kondrashov, 2018 [25]	Gross anatomy and radiology principles	Utilized surveys to assess student perspectives of ultrasound use in facilitating the understanding of gross anatomy and radiology principles
	McAllister et al., 2018 [26]	Obstetrics ultrasound lab	Fetal spine, stomach, heart chambers, and femur to determine fetal age and position using fetal ultrasound phantom
	Kondrashov et al., 2015 [10] Zoll et al., 2017 [16] Kondrashova & Kondrashov, 2018 [18] Kondrashova & Coleman, 2017 [27]	Clinical ultrasound course	Neck, heart, abdomen, gastrointestinal, pelvis, urinary system, lower extremities, and upper extremities vasculature. Musculoskeletal, upper limb, and lower limb. Ocular, echocardiography, focused assessment with sonography for trauma (FAST) examination, breast examination, needle-guided procedures, including central line placement, thoracentesis, and lumbar puncture, obstetrics, gynecology, lung, endocrine
	Kondrashova et al., 2020 [14] Zoll et al., 2017 [16]	Ultrasound needle-guided procedures lab	PICC and central line on thiel-embalmed cadaver and models
AZCOM	Brown et al., 2022 [11]	Eight-hour workshop labs on a clinical case with clinicians, trained sonographers, and anatomy faculty ultrasound questions on examinations	Lumbar puncture, upper limb anatomy of the shoulder, carpal tunnel, plantar fascia, calcaneal tendon, and tarsal tunnel
LECOM-BRADENTON	Syperda et al.,2008 [28]	Training with a radiologist or technologist	The abdominal region, the pelvic region in women, cardiac region, introduction to ultrasound instruments
NYIT-COM	Chan et al., 2017 [10]	OMM with pre-workshop videos	Lumbar spine anatomy
	De Vries et al., 2018 [7]	OMM	Shoulder landmarks, long head of biceps tendon, supraspinatus tendon, coracoid process, T1 transverse process
RVU-COM	Evans & Thiessen, 2019 [8]	System-based ultrasonography with video modules	Ultrasound physics, terminology, instrumentation, imaging modes, musculoskeletal, cardiac, thoracic, pulmonary, abdominal, head, neck, cardiovascular, FAST examination, resuscitative ultrasound
	Weston et al., 2020 [19]	Workshop	Tibia, radius, metacarpal bone fracture identification in formalin-embalmed cadavers
	Clason et al., 2021 [20]	Two hours consisting of didactic, practice, and assessment	Knee arthrocentesis using formalin-embalmed cadavers
	Loveless et al., 2022 [21]	10-minute training video and practice. Assessment with a skills test	Subclavian vein access using formalin-embalmed cadavers
TCOM	Miller et al., 2022 [12]	Faculty and near-peer one-hour training sessions	Not listed
TUCOM	Hendriksz et al., 2018 [13]	Ultrasound simulation program aligned with anatomy curriculum, small group skills labs, and assessments	Cardiac, blood vessels, vasculature, liver, kidneys, gallbladder, lungs, ocular, fetal, musculoskeletal, FAST examination
VCOM	German et al., 2022 [22]	One-hour peer training and assessment of the skill	Knee arthrocentesis with knee models
WVSOM	Goodcoff et al., 2019 [15]	Student-driven extracurricular sessions in PAL format	Basic lung examination, FAST examination, RUQ biliary examination, central venous catheter placement

In the review of the literature, there were several positive themes within the implementation of US inside of osteopathic curricula. There was also a myriad of unknowns that may permit broader research with regard to the pedagogical improvement of US instruction within these institutions.

Using the previously described eligibility criteria, our review revealed only 22 eligible sources out of the original 2,968 citations discovered. This indicates that most available literature was either beyond the scope of the study or was not focused enough for generalizable insights into the implementation of US on students’ performance in osteopathic preclinical medical education. For example, the ATSU article from Petty et al. [23] directly assessed student performance, while most other sources did not. This lack of uniformity between studies, and the small population that was evaluated, limited the ability to draw conclusions between the independent studies referenced. As a result, the ability to provide generalizable recommendations regarding US implementation is limited.

Only 10 out of 44 osteopathic institutions were shown within the literature to be actively measuring the impact of US instruction during the preclinical years (Rowan University School of Osteopathic Medicine recently published two papers on US that did not meet our inclusion criteria [29,30]). There may be several reasons for the low number of publications. Some institutions have pointed toward financial barriers and logistical issues as a concern for the implementation of US into their curriculum [7,11,19]. However, the cost of POCUS has become less of a barrier to implementation in undergraduate medical education, and this may be a reason why nine articles involving POCUS have been published in the last year and a half [11,12,20-22,26,28]. Self-directed education and peer instruction environments can further reduce the barrier to implementation [12]. Nevertheless, 33 of 44 osteopathic schools have adopted some form of US into their curricula [31]. Combining our results with the published data [29-31] suggests 23 osteopathic schools have made curricular changes without publishing outcome data.

Regardless of potential barriers, there is a growing opportunity for the expansion of US education in osteopathic undergraduate medical curricula. Analysis of this small body of literature indicates a positive perception by students who engage in US integrated curriculum [17,19]. It was also noted that successful implementation of US curricula promoted lasting impacts on the learning environment through medical student performances on standardized examination practices [18]. Given this and the marked success and adoption of ultrasonography in modern healthcare, the demand for US education in early medical training is increasing [5,10,14,24,25,28]. However, further study is needed with respect to integrating US into medical school curricula, and future investigations may provide additional insights into academic outcomes, such as COMLEX and USMLE pass rates, student class standing, and residency match rates.

## Conclusions

Overall, our review of the literature identified the need for further study regarding the use of US in preclinical course work as well as several introductory themes concerning US implementation into undergraduate preclinical osteopathic education. These themes included potential barriers to adopting the technology as well as instructional perceptions of its use in preclinical courses, such as anatomy or OMM. To increase the benefits of medical education, it may be necessary to include US in the anatomy curricula of undergraduate and graduate medical students. While there is still a great deal of exploration to be done within osteopathic institutions for the adoption of US in preclinical curricula, the perceived benefit for students seems to be high within current publications.
